# On-admission serum 25(OH)D level and mortality within one year in older patients

**DOI:** 10.1186/s12877-024-05166-z

**Published:** 2024-08-08

**Authors:** Nadav Abel, Felicia Stern, Sari Tal

**Affiliations:** 1 Geriatric Department, Hartzfeld Hospital at Kaplan Medical Center, Affiliated with the Hebrew University of Jerusalem, Rehovot, Israel; 2 The Robert H. Smith Faculty of Agriculture, Food and Environment, the Hebrew University of Jerusalem, Rehovot, Israel; 3 Acute Geriatrics Department, Kaplan Medical Center, Affiliated with the Hebrew University of Jerusalem, Rehovot, Israel

**Keywords:** Older adults, Hypovitaminosis D, Mortality, Risk factors

## Abstract

**Background:**

Mounting evidence suggests that vitamin D deficiency is associated with a higher risk of many chronic non-skeletal, age-associated diseases as well as mortality.

**Aim:**

To determine, in older patients aged ≥ 80, the prevalence of vitamin D deficiency and its association with comorbidity, laboratory tests, length of stay and mortality within one year from blood withdrawal on admission to acute geriatrics ward.

**Methods:**

We retrospectively surveyed electronic hospital health records of 830 older patients. The recorded data included patient demographics (e.g., age, sex, stay duration, readmissions number, death within one year from blood withdrawal on admission), medical diagnoses, laboratory results, including 25-hydroxyvitamin D [25(OH)D], and medications. We compared the characteristics of the patients who survived to those who died within one year.

**Results:**

On admission, in 53.6% patients, vitamin D levels were lower than 50 nmol/L, and in 32%, the levels were ≤ 35 nmol/L. Persons who died were likely to be older, of male sex, were likely to be admitted for pneumonia or CHF, were likely to have lower level of albumin or hemoglobin, lower level of vitamin D or higher vitamin B12 and higher level of creatinine, were also likely to have had a lengthier hospitalization stay, a greater number of hospitalizations in the last year, a higher number of comorbidities, to have consumption of ≥5 drugs or likely to being treated with insulin, diuretics, antipsychotics, anticoagulants or benzodiazepines. Higher age, male sex, on-admission CHF, higher number of drugs, lower albumin, higher vitamin B12, vitamin D < 50 nmol/L, and consumption of antipsychotics and anticoagulants – were predictors of mortality.

**Conclusion:**

Hypovitaminosis D is predictive of mortality in older patients within one year from hospitalization in the acute geriatric ward, but a causal relationship cannot be deduced. Nevertheless, older patients in acute care settings, because of their health vulnerability, should be considered for vitamin D testing. In the acutely ill patients, early intervention with vitamin D might improve outcomes. Accurate evaluation of mortality predictors in this age group patients may be more challenging and require variables that were not included in our study.

## Background

Vitamin D is an essential nutrient and a pro-hormone that regulates and mediates many physiologic processes, including bone metabolism, calcium homeostasis, extra-skeletal metabolism, cardiovascular homeostasis as well as immune functions [1–4]. Low serum vitamin D levels are frequent in older adults [5]. A combination of inadequate exposure to solar radiation and less than optimal dietary intake results in vitamin D deficiency [6].

Hypovitaminosis D reduces formation of the active form of vitamin D (1,25-dihydroxyvitamin D) at the tissue level [7]. The vitamin D receptor is found in most body tissues, and 1,25-dihydroxyvitamin D has a diverse array of biological functions ranging from anti-proliferative and anti-angiogenic effects to modulation of the immune system [4, 8]. This may be critical in mediating the beneficial pleiotropic functions of vitamin D, involving innate immunity, mucosal barrier, and endothelial function [7].

Vitamin D deficiency has been related to inflammation and immune dysfunction, which may be the potential reason for the increased susceptibility of the individual to severe infection [2]. In hospitalized very old subjects, lower levels of serum 25-hydroxy vitamin D [25(OH)D] are associated with a higher comorbidity burden [9]. Since there are vitamin D receptors in most tissues and cells in the body [10], mounting evidence suggests that vitamin D deficiency is associated with a higher risk of many chronic non-skeletal, age-associated diseases, including cancer, heart disease, diabetes mellitus, cognitive dysfunction, and dementia [1] as well as mortality [4, 6, 11, 12]. Some studies have also found an association between lower levels of vitamin D and frailty [5].

A multitude of association studies and meta-analyses have demonstrated the potential protective effects of vitamin D in reducing risk of cardiometabolic disorders, several types of cancers, autoimmune disorders, all-cause mortality, and other acute and chronic illnesses [13] An inverse association was observed between 25(OH)D and cardiovascular diseases, infections, glucose metabolism, cognitive disorders, and all-cause mortality [14]. Whether 25(OH)D is a marker of organ diseases is still under debate [9]. Though epidemiological evidence suggests that vitamin D deficiency is associated with increased mortality [12], the evidence is inconclusive, and the possible pathways remain unresolved [8]. Also, it is unclear if there are specific causes of death for which vitamin D might be important [12]. In addition, it is unclear whether low vitamin D levels independently contribute to mortality [15].

Our study aimed to determine, in older patients aged ≥ 80, the prevalence of vitamin D deficiency and its association with comorbidity, laboratory tests, length of stay and mortality within one year from blood withdrawal on admission to the acute geriatric ward.

## Methods

### Participants, design and procedures

We retrospectively surveyed electronic hospital health records of 830 older patients, aged ≥ 80 years, admitted between January 2015 and December 2017 from the emergency room to the acute Geriatrics Department at Kaplan Medical Center (a large community-based general hospital), Rehovot, Israel.

The recorded data included patient demographics (e.g., age, sex, stay duration, readmissions number, death within one year from blood withdrawal on admission), medical diagnoses, laboratory results, including 25-hydroxyvitamin D [25(OH)D], and medications. We retrieved the following data: admission diagnoses as well as comorbidities, including pneumonia, ischaemic heart disease (IHD), congestive heart failure (CHF), cerebrovascular accident (CVA), atrial fibrillation (AF), acute renal failure (ARF), diabetes mellitus, chronic renal failure (CRF), chronic obstructive pulmonary disease (COPD), dementia, obesity, malignancy, and falls. The drug types we retrieved were: statins, β-blockers, benzodiazepines, antipsychotics, antiplatelets, anticoagulants, insulin, antidiabetics, and proton pump inhibitors (PPI). We compared the characteristics of the patients who survived to those who died within one year from blood withdrawal on admission.

The study was approved by the Institutional Ethics Committee of the Kaplan Medical Center, Rehovot, Israel.

### Statistics

Statistical analysis was performed, using JMP 17.0 Pro software (SAS Institute Inc., Cary, NC). Patients who survived were compared to those who died within one year from on-admission blood withdrawal, for categorical and continuous characteristics. Univariate significance was established by Chi-Square tests for categorical characteristics and by t-tests for continuous characteristics. Variables with univariate significance of 0.10 or less were used for a stepwise logistic regression model using alpha = 0.10 to enter the model and alpha = 0.05 to leave the model. After employing this criterion, the association between the remaining variables and death were used in a multiple logistic regression model. Results are presented as odds ratios with 95% confidence limits and significance from the possibility OR = 1.

## Results

The mean age of the study population was 87.50 ± 4.87 years (range 80–112). Selected characteristics of the 830 older study subjects (61.4% females), divided according to vitamin D levels, are presented in Table 1. On admission, in 445 (53.6%) out of the 830 study subjects, vitamin D levels were lower than 50 nmol/L, and in 268 (32%) of the study subjects the levels were ≤ 35 nmol/L, 39% of the patients had low serum albumin, about 84% consumed ≥ 5 drugs, and about 52% consumed ≥ 10 drugs. Approximately 21% of the studied population were supplemented with vitamin D.

**Table 1:** Univariate analysis of selected characteristics of older persons, by vitamin D level (*N* = 830)

Characteristics	Vitamin D level (nmol/L)				
	≤ 35 (*n* = 268}	35-<50 (*n* = 177}	50–75 (*n* = 238}	> 75 (*n* = 147}	*P*
Age, years, mean ± SD	87.16 ± 5	87.14 ± 4.94	87.61 ± 4.98	86.86 ± 6.2	0.5983
Male, n (%)	110(40.7)	73(41.7)	77(32.2)	60(41.1)	0.1209
Female, n (%)	158(59.3)	104(58.3)	161(67.8)	87(58.9)	
Mortality within one year, n (%)	71(26.3)	37(21.1)	29(12.1)	27(18.5)	0.0007
Length of hospital stay, mean ± SD	5.23 ± 3.87	5.06 ± 3.36	5.33 ± 4.1	4.74 ± 3.28	0.4988
Number of previous hospitalizations, mean ± SD	1.92 ± 3	1.7 ± 2.24	1.53 ± 2.07	1.97 ± 2.53	0.2603
Number of comorbidities, mean ± SD **Drugs**	7 ± 3.68	5.06 ± 3.36	5.33 ± 4.1	4.74 ± 3.28	0.2232
			n (%)		
≥ 5	218(80.7)	145(82.9)	207(86.6)	131(89.7)	0.0623
≥ 10	134(49.6)	88(50.3)	122(51.0)	89(61.0)	0.1308
Insulin	35(13)	12(6.9)	14(5.9)	14(9.6)	0.0295
Antidiabetic	95(35.2)	46(26.3)	69(28.9)	29(19.9)	0.0080
Statins	137(50.7)	97(55.4)	133(55.6)	96(65.8)	0.0321
Benzodiazepines	74(27.4)	66(37.7)	96(40.2)	62(42.5)	0.0035
Antipsychotics	35(13.0)	29(16.6)	32(13.4)	20(13.7)	0.7415
Vitamin D supplement	23(8.5)	37(21.1)	59(24.7)	59(13.7)	0.0000
Antiplatelets	128(47.4)	95(54.3)	117(49.0)	68(46.6)	0.4632
Anticoagulants	80(29.6)	45(25.7)	63(26.4)	59(40.4)	0.0165
PPI	127(47)	76(43.4)	112(49.9)	65(44.5)	0.8569
**Admission Diagnosis**					
			n(%)		
CVA	13(4.8)	10(5.7)	17(7.1)	5(3.4)	0.4321
IHD	16(5.9)	7(4.0)	16(6.7)	10(6.8)	0.6231
ARF	34(12.6)	13(7.4)	28(11.7)	14(9.6)	0.3107
CHF	45(16.7)	26(14.9)	50(20.9)	29(19.9)	0.3589
Falls	32(11.9)	13(7.4)	19(7.9)	17(11.6)	0.2688
Pneumonia	39(14.4)	25(14.3)	41(17.2)	26(17.8)	0.6956
UTI	25(9.3)	20(11.4)	30(12.6)	17(11.6)	0.6752
**Comorbidities**					
	n (%)				
CVA	43(15.9)	22(1.26)	26(10.9)	22(15.1)	0.3603
Diabetes	119(44.1)	57(32.6)	82(34.3)	44(30.1)	0.0127
CRF	72(26.7)	47(26.4)	63(26.4)	39(26.7)	0.9995
CHF	33(12.2)	19(10.9)	26(10.9)	13(8.9)	0.7768
IHD	92(34.1)	63(36.0)	81(33.9)	66(45.2)	0.1087
COPD	47(17.4)	25(14.3)	40(16.7)	17(11.6)	0.3937
Dementia	46(17.0)	30(17.1)	36(15.1)	28()19.2)	0.7713
Falls	15(5.6)	9(5.1)	15(6.3)	7(4.8)	0.9289
Osteoporosis	35(13.0)	31(17.7)	60(25.1)	44(30.1)	0.0001
Obesity	15(5.6)	16(9.1)	29(12.1)	13(8.9)	0.0704
Malignancy	42(15.6)	27(15.4)	42(17.8)	22(15.1)	0.8947
**Laboratory tests**					
Albumin, mean ± SD	3.38 ± 1	3.49 ± 0.44	3.52 ± 0.45	3.6 ± 0.47	0.0003
Sodium, mean ± SD	137.76 *±* 5.02	138.60 *±* 4.97	138.05 *±* 6.15	137.76 *±* 5.30	0.2345
Calcium, mean ± SD	8.59 ± 1	8.71 ± 0.67	8.86 ± 0.88	8.81 ± 0.64	0.0009
Vitamin B12 (*n* = 660), mean ± SD	390.96 ± 234.32	409.6 ± 255.35	450.179 ± 243.73	506.922 ± 291.29	0.0005
Low vitamin B12, n (%)	35(16.8)	23(17.2)	15(7.5)	6(5.2)	0.0005
High Vitamin B12, n (%)	23(11.0)	20(14.9)	31(15.4)	28(24.1)	0.0246
Creatinine, mean ± SD	1.29 ± 1	1.25 ± 0.84	1.24 ± 0.76	1.26 ± 0.71	0.9393
High creatinine, n (%)	130(52.2)	78(48.5)	113(50.7)	73(55.3)	0.6878
Folic acid, mean ± SD	18.73 ± 12	21.2 ± 12.55	23.22 ± 12.41	23.89 ± 11.68	0.0016
Low folic acid, n (%)	80(46.0)	39(34.8)	35(22.0)	16(19.3)	< 0.0001
Vitamin D, mean ± SD	22.89 ± 7.26	41.93 ± 4.28	61.48 ± 6.82	93.31 ± 16.83	< 0.0001

Low vitamin B12, < 200 pmol/L; high vitamin B12, > 666 pmol/L; Low albumin, < 3.5; folic acid, 13.4–56.5 mmol/L; low folic acid, < 13 mmol/L; high creatinine, > 1

UTI – urinary tract infection; COPD – chronic obstructive pulmonary disease; CHF – congestive heart failure; CVA – cerebrovascular accident; CRF – chronic renal failure; IHD – ischemic heart disease; ARF – Acute renal failure

Within one year, 164 persons (19.8%) died, of whom 49.4% were women (Table 2). Compared with persons who survived, persons who died were likely to be older, of male sex, were likely to be admitted for pneumonia or CHF, were likely to have lower level of albumin or hemoglobin, lower level of vitamin D or higher vitamin B12 and higher level of creatinine, were also likely to have had a lengthier hospitalization stay, a greater number of hospitalizations in the last year, a higher number of comorbidities, to have consumption of ≥5 drugs or likely to be treated with insulin, diuretics, antipsychotics, anticoagulants or benzodiazepines. Mortality within one year from on-admission blood withdrawal, was higher among patients with low albumin levels as well as patients with lower vitamin D and lower or higher vitamin B12 levels.

**Table 2:** Univariate analysis of selected characteristics of the study participants, by mortality within one year (*N* = 830)

Characteristic	Died *n* = 164	Survived *n* = 666	*P*
Age, years (mean ± SD)	87.96 ± 5.77	93.13 ± 3.06	0.0496
Female sex, n (%)	81(49.4)	429(64.4)	
Male sex, n (%)	83(50.6)	237 (35.6)	0.0005
Length of hospital stay (days), mean ± SD	6.11 *±* 4.60	4.90 *±* 3.45	0.0002
Number of hospitalizations in last year, mean ± SD	2.27 ± 2.41	1.65 ± 2.46	0.0037
Number of comorbidities, mean ± SD	7.90 ± 3.89	6.80 ± 3.52	0.0005
Number of drugs, mean ± SD	14.01(7.95)	10.87(7.68)	0.0000
**Admission Diagnosis**			
	*n* (%)		
Pneumonia	36 (22.0)	95 (14.3)	0.0194
CHF	41(25.0)	109(16.4)	0.0126
IHD	7 (4.3)	42 (6.3)	0.3035
CVA	6(3.7)	39(5.9)	0.2443
ARF	20(12.2)	69(10.4)	0.5025
AF	10(6.1)	42(6.3)	0.9210
Falls	17(10.4)	64(9.6)	0.7715
**Comorbidities**			
	*n* (%)		
CVA	25 (15.2)	88 (13.2)	0.5021
Diabetes Mellitus	65(39.6)	237 (35.6)	0.3365
CRF	53 (32.3)	168 (25.2)	0.0699
CHF	29 (17.7)	62 (9.3)	0.0036
IHD	79 (48.2)	223 (33.5)	0.0005
COPD	28 (17.1)	101 (15.2)	0.5496
Dementia	44 (26.8)	96 (14.4)	0.0003
Falls	8(4.9)	38(5.7)	0.6736
Obesity	12(7.3)	61(9.2)	0.4463
Malignancy	29(17.7)	104(15.6)	0.5222
**Drugs**			
	*n* (%)		
≥ 5	154 (93.9)	547 (82.1)	0.0000
≥ 10	107(65.2)	326(48.9)	0.0002
Insulin	24 (14.6)	51 (7.7)	0.0083
Antidiabetics	117(71.3)	474(71.2)	0.9656
Diuretics	104 (63.4)	304 (45.6)	0.0000
Benzodiazepines	73 (44.5)	225 (33.8)	0.0111
Antipsychotics	39 (23.8)	77 (11.6)	0.0001
Antiplatelets	86(52.4)	322(48.3)	0.3479
Anticoagulants	71(43.3)	176(26.4)	0.0000
Vitamin D, supplemented	41(25)	137(20.6)	0.2222
**Laboratory tests**			
Albumin, (*n* = 753), mg/dL, mean ± SD	3.2 ± 0.54	3.55 ± 0.46	0.0000
Low Albumin (*n* = 327), n (%)	102 (68.5)	225 (37.3)	0.0000
Creatinine, mg/dL, mean ± SD	1.6 ± 0.98	1.21 ± 0.72	0.0001
High creatinine, (*n* = 170), n (%)	96 (63.2)	298 (48.6)	0.0012
Vitamin D, nmol/L, mean ± SD	46.06 ± 27.75	51.58 ± 26.17	0.0171
Vitamin D < 50 mmol/L, n (%)	108(65%)	337(50.6%)	0.0004
Folic acid (*n* = 528), mmol/L, mean ± SD	22.13 ± 13.05	21.26 ± 12.09	0.5911
Low folic acid, n (%)	36(37.5%)	134(31)	0.2235
Vitamin B12 (*n* = 660), pmol/L, mean ± SD	507.12 ± 271.48	415.02 ± 247.77	0.0002
High vitamin B12 (*n* = 102), n (%)	32 (24.6)	70 (13.2)	0.0149
Low vitamin B12 (*n* = 79), n (%)	10(7.7)	69 (13.0))	0.0791
Haemoglobin, (*n* = 695), g/dL, mean ± SD	11.12 ± 1.90	11.49 ± 1.44	0.0282
Sodium (*n* = 765), mg/dL, mean ± SD	139.17 ± 6.95	137.65 ± 4.92	0.0019
Calcium (*n* = 755), mg/dL, mean ± SD	8.59 ± 0.75	8.77 ± 0.75	0.0078

Low vitamin B12, < 200 pmol/L; high vitamin B_12_, > 666 pmol/L; Low albumin, < 3.5; High creatinine, > 1; folic acid, 13.4–56.5 mmol/L; low folic acid, < 13

ARF – acute renal failure; COPD – chronic obstructive pulmonary disease; CHF – congestive heart failure; CVA – cerebrovascular accident; CRF – chronic renal failure; IHD – ischemic heart disease

According to multiple logistic regression analysis (Table 3), the independent variables – higher age, higher number of drugs, lower level of albumin, higher level of vitamin B12, vitamin D < 50 nmol/L, male sex, antipsychotics, anticoagulants, and on-admission diagnosed CHF – were predictors of mortality within one year. The significant mortality predictors, found in the study, explain about 21% of the possible predictors of mortality within one year from on-admission blood withdrawal.

**Table 3:** Risk factors for mortality within one year by multiple logistic regression analysis

Parameter	Odds Ratio	95% Confidence Interval	*P*
Number of hospitalizations in the last year	0.886	0.777–1.010	0.0698
Age	1.046	1.003–1.090	0.0343
Male sex	2.445	1.548–3.863	0.0001
Number of drugs	1.076	1.035–1.118	0.0002
Vitamin B12	1.001	1.001–1.002	0.0009
Vitamin D < 50	2.282	1.420–3.666	0.0006
Albumin	0.231	0.141–0.378	< 0.0001
Antipsychotics	2.035	1.132–3.658	0.0175
Anticoagulants	2.587	1.587–4.215	0.0001
CHF at admission	1.855	1.082–3.181	0.0247
Dementia	1.434	0.822–2.503	0.2045

R^2^ = 0.2137

CHF – congestive heart failure; CVA – cerebrovascular accident

## Discussion

Our study deals with older adults ≥ 80 years old. We studied the risk factors of mortality within one year from blood withdrawal on admission to the acute geriatric ward. A higher percentage of men than women died, quite similarly to the findings of some other studies [16–18] and in contrast to other studies [19, 20]. Higher age was an independent risk factor of mortality. The risk of death was higher by about 5% in older subjects, although surprisingly, the mean age of the older adults, who survived was higher (93.13 ± 3.06) than that of those who died (87.96 ± 5.77). It can be explained by a higher proportion of women among the survivals (52% vs. 29%) in whom life expectancy is higher.

In our study, on-admission serum 25(OH)D levels below 50 nmol/L, were determined in 53.6% of the study population. Serum 25(OH)D is generally accepted as first choice measurement of vitamin D status, and it is used to classify vitamin D. The Endocrine Society Guidelines classify 25(OH)D levels below 50 nmol/L as deficient and levels of 75 nmol/L as sufficient, whereas the Institute of Medicine (IOM) suggested vitamin D deficiency to be indicated by 25(OH)D levels below 30 nmol/L and a level of 50 nmol/L to be sufficient [8].

Hypovitaminosis D is frequent in older subjects, ranging in occurrence from 50 to 90%, depending on the definition used and the setting [9]. Studies conducted in Australia revealed a higher risk of vitamin D deficiency in older people, who were hospitalized or residing in residential facilities [5].

Vitamin D is essential for bone mineralization, but a growing body of evidence points at a broader role [4, 8]. Vitamin D receptors are ubiquitous in the human body, and while the endocrine effects of vitamin D are well recognized, less appreciated are the autocrine and paracrine effects (antimicrobial and immunomodulatory) [6].

In hospitalized very old subjects, a higher comorbidity burden is associated with lower 25(OH)D serum levels [9]. Vitamin D deficiency has been found to be associated with mortality, and several diseases ranging from cardiovascular disease to autoimmune diseases and liver diseases [6, 8]. However, it is unclear whether this is explained by reverse causation, and if there are specific causes of death for which vitamin D might be important [12].

Despite an upsurge in medical research, the literature on determinants of survival in geriatric inpatients is scarce. Most of the studies, dealing with vitamin deficiency and its association with morbidity and mortality, have been conducted in older adults living in the community. In our study, vitamin D insufficiency (levels of < 50 nmol/L) was an independent risk factor of mortality, and increased mortality risk by 2.28. A retrospective study by Dudenkov et al. [14] reported a statistically significant inverse relationship between serum 25(OH)D levels and mortality in 11,022 white and nonwhite community patients registered in the Rochester Epidemiology Project. In an observational study of osteoporotic women aged 75 and older, 25(OH)D levels of less than 50 nmol/L were associated with greater all-cause mortality for up to 10 years. In that study, in women with vitamin D levels < 50 nmol/L, mortality risk in the low category was almost doubled twice than the high category, and remained higher even after adjustment for smoking and comorbidities, and was highest when additionally adjusted for osteoporosis, HR = 2.1 [15]. In a prospective cohort study of Australians older men, the risk of death was greater in those with vitamin D levels < 50 nmol/L than in those with a serum 25(OH)D level of 75 nmol/L or greater (adjusted HR = 1.42). In that study hypovitaminosis D is an independent predictor of all-cause mortality, regardless of frailty status or other comorbidities. The association between vitamin D and mortality is not solely dependent on the occurrence of frailty. Vitamin D may be a contributor in the development of frailty, but frailty is unlikely to play a major role in the association between vitamin D and all-cause mortality [5].

Very few studies have demonstrated the interaction between serum 25(OH)D and frailty in their association with mortality, and they were conducted in older adults in the community. One possible explanation for the relationship between serum 25(OH)D and frailty in their association with mortality is the fact that older adults are more likely to suffer from frailty, which increases their risk of falling, which in turn, might mediate between vitamin D deficiency and death. Since the study was performed in the oldest old patents, we presume that many of the study patients were frail. But no statement can be made about the frailty of these patients because no indicative data on frailty was found in the electronic hospital health records. A clear independent relationship between vitamin D status with frailty and mortality has yet to be established [5], through further future studies.

There are few studies on severely ill and hospitalized patients with malnutrition – a condition that puts patients at particular risk for adverse clinical outcomes. Vitamin D deficiency is highly prevalent in the population of malnourished inpatients and is negatively associated with mortality [21]. In a study in critically ill patients, a longer survival was observed in vitamin D sufficient patients [22]. A systematic review and meta-analysis, which investigated effects of vitamin D deficiency on adverse medical outcomes across different medical inpatient populations, found a stepwise increase in mortality of 3.4%, 5.6%, and 8.7% in patients with vitamin D sufficiency, insufficiency (25 to 50 nmol/L), and severe deficiency, respectively, and vitamin D deficiency increased 30-day mortality by 1.70 for vitamin D insufficient levels compared to sufficient levels (Vit D ≥ 50 nmol/L) [23]. In a prospective cohort Swiss study, inpatients with vitamin D levels, had an increased 180-day mortality rate of 29.9% compared to 23.1% in patients with no deficiency, and vitamin D deficiency increased mortality risk by 1.42 [21]. A comparison between mortality in deficient/insufficient subjects and in those with no deficiency emphasizes the importance of vitamin D in reducing mortality in the oldest old adults.

In the present study, additional factors were independent risk factors of mortality.

Hypoalbuminemia is a prognostic risk factor of mortality among older adults [10, 24]. Low levels of albumin are associated with worse recovery following acute pathologies [25]. Higher albumin levels, in our study, had a protective effect, correspondingly to other studies [24, 26]. Serum albumin plays a vital physiologic role in health maintenance for many organs. Hypoalbuminemia might be an indicator of malnutrition, which explains the bad prognosis [24, 27]. Hypoalbuminemia, in the present study, was associated with lower serum vitamin D levels, deficiencies that might eventually deteriorate to death, especially when being independent risk factors of mortality. Concomitant conditions of hypoalbuminemia and lower vitamin D levels may prompt to look for a high-risk group of geriatric patients, who could be targeted for more careful and closer follow-up and intervention for an extended time.

In our study, a higher rate of older adults with on-admission CHF died, and the risk of mortality among them was higher by about 1.9. In a study in hospitalized patients aged *≥* 80 years with acute myocardial infarction, on-admission left ventricular ejection fraction *<* 40% predicted 1-year mortality [28]. Comorbid CHF in patients aged ≥ 90 hospitalized in acute geriatric ward, was an independent risk factor of in-hospital mortality [29].

In the present study, a higher proportion of older adults with high vitamin B12 levels died, and high vitamin B12 level was a significant predictor of mortality. In many studies performed on the aged, the focus is usually on detecting vitamin B12 deficiency [30]. In contrast, less is known about the meaning of high vitamin B12 levels. High vitamin B12 levels, as a predictor of increased mortality in malignancies and liver disease, have been already documented [31, 32]. Likewise in our study, in several studies, high vitamin B12 levels, in older patients suffering from acute and chronic diseases except for malignancy, were associated with higher mortality [33–39].

Older people are prescribed a greater number of medications which may be inappropriate, fueling the cycle of comorbidity, disability, hospitalization, nursing home placement, and mortality [40]. In the present study, a higher percentage of older adults, who consumed ≥ 5 drugs, died, and a higher number of drugs consumed was found to be an independent risk factor of mortality.

In our study, the risk of mortality, in subjects who consumed antipsychotics, was higher by slightly more than twice. Antipsychotic use was an independent risk factor of mortality, correspondingly to Tal’s finding found in oldest old patients within one year after discharge from acute geriatric ward [41]. Also, in some other studies antipsychotics consumption enhanced mortality [42, 43]. Antipsychotic medications are used to treat and manage symptoms of many psychiatric disorders [44]. There are several likely mechanisms by which antipsychotics may increase the risk of death. Antipsychotics may prolong the QT interval, predisposing patients to arrhythmias and sudden cardiac death. Additionally, sedation and accelerated cognitive decline, caused by antipsychotics, may increase the risk of aspiration and choking, especially in patients with dementia [45].

In our study, a higher percentage of subjects, treated with anticoagulants, died, and anticoagulant treatment increased the risk of mortality by about 2.6. Although it was shown in several studies, that anticoagulant therapy clearly outweighs the risk of heavy bleeding, even in the older adults [46–48], the clinician should probably consider treating with caution older patients with many comorbidities, including falls.

Our study has all the disadvantages of a retrospective observational study. We did not have all the details that may have been influencing mortality in the older adults within one year after on-admission blood withdrawal, because they were not found in the patients’ electronic data. Consequently, the significant independent variables (age, male sex, number of drugs, on-admission CHF, low albumin, vitamin B12, vitamin D < 50, antipsychotics or anticoagulants use) comprise only part of mortality predictors. Some other unknown variables might explain the remainder.

Our study strength lies in its sample’s relatively large size. Since the study focuses on mortality predictors in the ≥ 80-year adults within one year from on-admission blood withdrawal, we would like to point out that we have contributed to the medical knowledge concerning this population of older adults, whose proportion in the geriatric population is recently significantly increasing.

## Conclusion

Hypovitaminosis D is predictive of mortality in patients within one year from hospitalization in the acute geriatric ward. Although we have shown a significant association between the vitamin level and mortality, a causal relationship cannot be deduced. Nevertheless, older patients hospitalized in acute care settings, because of their health vulnerability, should be considered for vitamin D testing. In the acutely ill patients, early intervention with vitamin D might improve outcomes. Accurate evaluation of mortality predictors in this age group patients may be more challenging and require variables that were not included in our study. Future research is warranted.

## Data Availability

The datasets used during the current study are available from the corresponding author on reasonable request.

## Abbreviations

25(OH)D: 25-hydroxyvitamin D
IHD: Ischaemic heart disease
CHF: Congestive heart failure
CVA: Cerebrovascular accident
AF: Atrial fibrillation
ARF: Acute renal failure
CRF: Chronic renal failure
COPD: Chronic obstructive pulmonary disease
PPI: Proton pump inhibitors
OR: Odds Ratio
HR: Hazard Ratio
